# Coronavirus vaccination rates in cultural areas in Germany

**DOI:** 10.1017/S0950268822000085

**Published:** 2022-01-17

**Authors:** Claudia Spahn, Anna Maria Hipp, Bernhard Richter, Manfred Nusseck

**Affiliations:** Freiburg Institute for Musicians’ Medicine, University Medical Center Freiburg, University of Music Freiburg, Medical Faculty of the Albert Ludwigs University Freiburg, Freiburg Center for Research and Teaching in Music, Freiburg, Germany

**Keywords:** Coronavirus, cultural events, vaccination rates

## Abstract

Vaccination is a significant preventive measure to contain the coronavirus disease-2019 (COVID-19) pandemic. Vaccination rates can provide useful information on the potential spread of infection in a given population. In this study, vaccination rates and attitudes towards vaccination in cultural sectors, specifically the music sector, have been investigated. In total, 4341 persons in four different areas, including visitors to performances of classical music and musicals, as well as professional and amateur musicians, have participated in this survey. Results show rates of 86% recovered from the COVID-19 virus or vaccinated at least once, with 54.5% fully vaccinated. These vaccination rates were considerably higher compared to the general population. An attitude of hesitation towards vaccination found in 6.4% of those sampled was half that of the general population. These findings drawn from a large sample indicate that in the field of music a high vaccination rate is to be found, as well as a low rejection rate of vaccination on the part of the audience and performers. The results can be used to provide insights into the vaccination status to be found at cultural events and, importantly, to assist in consideration of whether cultural events should be permitted to continue under pandemic circumstances.

## Introduction

The impact of coronavirus disease-2019 (COVID-19) has had a tremendous effect on cultural life. Music concerts, theatres, festivals and nearly all other kinds of live performances have been restricted by the government in response to the present pandemic. The same holds true for rehearsals with large groups of musicians, i.e. for singers and brass instrumentalists. During a period this year of dropped incidence rates (spring and summer 2021), some cultural areas reopened with limited access. To enable potential further openings, the infection situation should be investigated, as well as the level of vaccination in those participating in such events. However, no studies exist yet that focus specifically on vaccination rates among participants in cultural events.

Current vaccines against COVID-19 have been shown to be highly effective [1–4]. These studies have shown that vaccination dramatically reduces the spread of the virus. It is therefore important to achieve a high rate of vaccination among people to reduce the incidence of infection [5]. Above a certain threshold of the proportion vaccinated in the population, general immunity can be achieved, known popularly as herd immunity [6]. Models indicate that herd immunity may be reached if at least 66% of the population has been vaccinated [7]. Such high rates of vaccination are currently found in certain occupational groups, such as healthcare workers with rates between 72.9% [8] and 89% [9].

However, the vaccination rates in the general population are normally considerably lower. At the time of the present study (June/July 2021), the epidemiological situation with regard to vaccination rates in Germany indicated that approximately 55% of the population had been vaccinated at least once and approximately 37% were fully vaccinated [10].

On the other hand, the effectiveness of vaccination depends on people's willingness to be vaccinated. Vaccine hesitancy can limit the benefits of vaccination in reducing the spread of the virus. The percentage of those reluctant to be vaccinated varies by country [11] but it also changes over time as long as the pandemic persists. In Germany, a survey from December 2020 with more than 2000 participants found that 33% were unwilling to be vaccinated [12]. In summer 2021 this amount fell drastically, with percentages at 6.1% for those undecided and 6.3% for those opposed to the vaccine [13].

In the cultural sector, it was expected to find vaccination rates similar to the general population.

According to the state government corona regulations [14] for participation at cultural events, it is mandatory to be either completely immunised through a certified recovery from a COVID-19 infection, through a full vaccination (the second shot must be at least 2 weeks prior), or demonstrably to be able to report a negative test (taken within less than 24 h).

Therefore, it seems of great importance to have the vaccination rates in the various branches of culture.

The goal of this study was to evaluate vaccination rates in the cultural sector. For this, the branch of music was specially chosen. A distinction was made between visitors to music events and actively participating musicians. In addition, the music sector was further subdivided into professional and amateur branches of activity. This resulted in four focus areas of 1. attendees at professional music events, 2. attendees at amateur live music events, 3. professional musicians and 4. amateur musicians. Participants were asked to indicate their vaccination status. The premise upon which the study is built is that knowledge of the general distribution of vaccinated people in cultural segments will provide insights into possible pandemic-related risks attendant upon reopening music events.

## Materials and methods

### Study procedure

The study was conducted in June and July 2021. It used online surveys and print versions of the questionnaire described below. On the title page of the print version and on the first page in the online survey, participants were informed that the questionnaire was anonymous and that participation was voluntary. By filling in the questionnaire, they agreed to participation in the study and to the scientific use of their data. The study had received ethical approval by the Ethics Committee of the University Medical Center Freiburg.

At different music events, the questionnaire was distributed to visitors with the request to fill it out. The specific locations of this study were part of a pilot project to investigate the effects of the pandemic on various cultural sectors. The classical music events were four operas and five ballets in south-west Germany (Baden-Württemberg) performed at one opera hall and one theatre respectively. Participants could decide which version, i.e. online or print, they prefer. The amateur music events were five musicals performed by amateur performers at an outdoor stage in south-west Germany (Baden-Württemberg). Only print versions of the questionnaire were used here. All events were presented in renowned venues where the audiences bought tickets for the events.

The musicians in the professional sector were music students and music teachers at the University of Music Freiburg. They were invited via email to participate in the online survey. The musicians in the amateur sector were recruited from 21 brass ensembles and brass band associations in south-west Germany (Baden-Württemberg). They were asked in rehearsals to participate in the study and to fill in the online survey. In all situations, the applicable COVID-19-related regulations were observed.

### Questionnaire

The questionnaire consisted of general questions about age (through age group categorisation) and gender, and specific questions about vaccination status. First, it asked if the person had contracted a COVID-19 infection (yes/no). The next question asked whether the person had been vaccinated (yes/no). If answered in the affirmative, participants were asked to indicate whether they were fully vaccinated, i.e. two shots (or one with J&J). If they were not vaccinated, they were asked if they had already made an appointment for vaccination. If not, they were asked to indicate whether they were in principle willing to be vaccinated, or if they were either undecided or dismissive about being vaccinated. The latter two answers, when responded to in the affirmative, were also expanded upon by questions concerning those respondents' reasons for not wanting to be vaccinated.

The rates and distribution data for persons in the cultural areas were compared with vaccination rates of the public at the same time. For that, the rates of the Robert-Koch-Institute (RKI) as reported in the COVID-19 Vaccination Monitor ‘COVIMO’ were drawn upon [13]. These rates represent the distribution within the general population of Germany at the time of the study.

### Statistics

The statistical analyses were performed using SPSS version 27 (IBM, Chicago, IL). The data were summarised by using descriptive statistics. Categorical data were compared using cross tables and Pearson's *χ*^2^ tests. The level of significance was set at *P* = 0.05.

## Results

A total of *N* = 4341 persons took part in this survey. The sample consisted of 58.1% female, 41.7% male, and 0.2% diverse. The group of visitors showed a higher proportion of females (in classical music events 69.8% and in amateur music events 60.5%). In contrast, the group of musicians were pretty much equally distributed between female and male musicians (female professional musicians: 47.0%; female amateur musicians: 47.4%).

In terms of age distribution, the largest groups were shown to be 18 to 26 years (21%), 46 to 55 years (18%) and 56 to 65 years (20%). The smallest age group was that of >80 years (3.6%). The other age groups had similar sizes of about 10%. There was a significant distribution difference in the age groups between the focus areas (*χ*^2^ = 1726.3; *P* < 0.001), with older participants more heavily represented in the group of visitors to classical music events and the younger participants more represented in the group of the professional musicians.

**Fig. 1.**
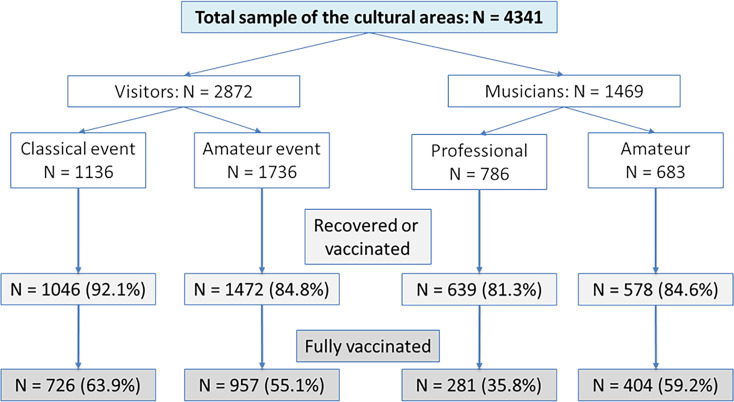
Vaccination rates of the different samples in the cultural sub-divisions.

Of the total sample, 86% reported being recovered or at least vaccinated once and 54.5% were fully vaccinated (Fig. 1). The share of recovered persons was in total 4.3%, of which 2.0% had not received full coverage with the second dose. Vaccinated and recovered persons were analysed together as they have similar immunisation effects.

There were significant differences in the vaccination rates between the cultural sub-divisions (*χ*^2^ = 180.1, *P* < 0.001). The highest rates of recovered and vaccinated persons were found in the group of visitors to classical music events with 92.1% recovered or at least vaccinated once and 63.9% fully immunised. The lowest rates were reported in the group of professional musicians (81.3% and 35.8% respectively).

**Fig. 2.**
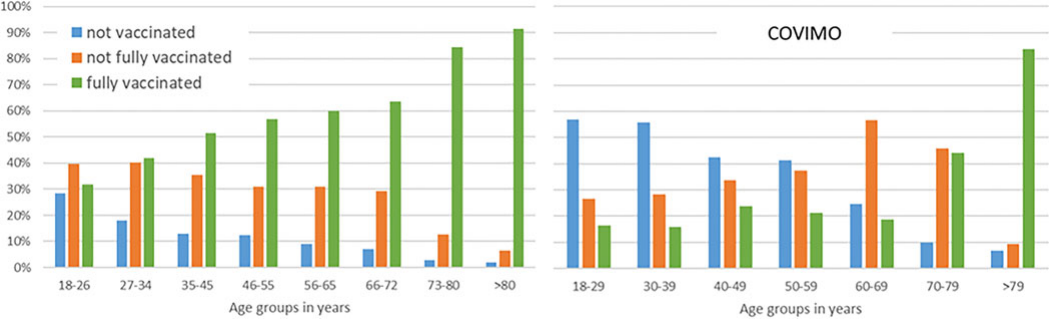
Vaccination rates by age groups (left: findings in this study, *N* = 4341; right: general population in Germany [13], *N* = 3004).

The distribution of the vaccination rates within each age group is shown in Figure 2. The proportion of participants who were recovered or vaccinated at least once was lowest for the youngest age group (18–26 years; 71.4%). All other age groups were above 80%. The share of fully vaccinated participants increased steadily across the age groups from 31.8% in the age group 18–26 years to the highest proportion in the age group >80 years with 91.5%.

For comparison purposes, the vaccination rates of the general population [13] are presented in Figure 2 in the column to the right of the rates revealed in this study. In every age group, the proportion of fully vaccinated participants in this study was approximately twice as high as in the general population, with the exception of the participants older than 79 years of age. The proportion of unvaccinated participants in this study was below 30% in the age group 18–26 and even lower than 20% for all other age groups. In the general population, the proportion of unvaccinated persons was lower than 30% only in the age groups over 60 years of age. The share of unvaccinated persons was more than twice as high as the share in this study for all age groups.

The overall proportion of participants in this study who showed reluctance to receive a vaccine was 6.4%. This amount can be divided into participants who were undecided about getting vaccinated (*N* = 143; 3.3%) and participants who declined vaccination (*N* = 133, 3.1%). Both proportions were significantly lower compared to the general population [13] of undecided persons (6.1%; *χ*^2^ = 32.8, *P* < 0.001) and of vaccination deniers (6.3%; *χ*^2^ = 43.1, *P* < 0.001). The reasons for vaccination hesitation were mainly the fear of side effects, uncertainty concerning the vaccines in the long-term, or that they simply refuse any kind of vaccination whatsoever.

## Discussion

In this study, the vaccination status of the population in different segments of music was evaluated. The results showed very high vaccination rates in all areas of music culture, indeed considerably higher than comparable rates in the wider population. When looking at the German population as a whole, a similar proportion of vaccinated and recovered persons can be found in healthcare workers [8, 9] and teachers [13].

The amount of vaccinated and recovered persons in the segments of culture found in this study exceeded the amount in the general population in all age groups. In Germany, there has been no compulsory vaccination to date, although specific groups of the population have been prioritised for vaccination, including older persons. As the attendees of cultural events are on average middle-aged and above, this factor may help explain the high vaccination rates found in the research. However, the results showed that even in younger age groups the vaccination rates were unusually high.

In particular, the reluctance to be vaccinated was very low compared to the general population. This indicates a high willingness for vaccination among those active in music culture, something very important in times of free access to vaccines. The findings also indicate that those persons involved in music culture, whether participants or audience, largely care about their safety and attend music events only with a certain degree of immunisation already in place.

One drawback of the study, however, is that it was regionally limited to the south-west of Germany. Given that the current incidence and vaccination rates across Germany show considerable differences between regions, it is thus less possible to generalise in a meaningful way from the findings, in a way that would apply to the whole of Germany. Nevertheless, we can assume that it is roughly similar in other regions specifically in the cultural activity of the type covered in this study.

In conclusion, the available data on a large sample of audiences in the field of music, classical and otherwise, as well as among active musicians in both the professional and amateur fields, show a high vaccination rate and willingness to be vaccinated in the cultural sector of music. Judging by the results of the present study, it can be expected that more than 90% of participants in the cultural sector will eventually receive full vaccination. This provides a very good starting point for considerations as to whether to keep cultural events and activities running, regardless of the prevailing pandemic situation. Moreover, it is also important that the necessary precautions for audiences, such as ventilation measures, wearing a mask and maintaining social distancing, be continued, to assess and ensure safe practices at cultural events. For musicians, specific risk-reducing measures have been developed and outlined by our working group [15].

## Data Availability

The data can be requested by the corresponding author.
